# Recyclable Superhydrophobic Surface Prepared via Electrospinning and Electrospraying Using Waste Polyethylene Terephthalate for Self-Cleaning Applications

**DOI:** 10.3390/polym15183810

**Published:** 2023-09-18

**Authors:** Taegyun Kim, Man Gyu Song, Kanghyun Kim, Hyungkook Jeon, Geon Hwee Kim

**Affiliations:** 1 Department of Mechanical Engineering, Chungbuk National University (CBNU), 1 Chungdae-ro, Seowon-gu, Cheongju-si 28644, Republic of Korea; 2 School of Mechanical Engineering, Chungbuk National University (CBNU), 1 Chungdae-ro, Seowon-gu, Cheongju-si 28644, Republic of Korea; 3 Department of Mechanical Engineering, Pohang University of Science and Technology (POSTECH), 77 Cheongam-Ro, Nam-Gu, Pohang 37673, Republic of Korea; 4 Department of Manufacturing Systems and Design Engineering (MSDS), Seoul National University of Science and Technology (SEOULTECH), 232 Gongneung-ro, Nowon-gu, Seoul 01811, Republic of Korea

**Keywords:** electrospinning, electrospraying, PET (polyethylene terephthalate), recycling, superhydrophobic

## Abstract

Superhydrophobic surfaces, i.e., surfaces with a water contact angle (WCA) ≥ 150°, have gained much attention as they are multifunctional surfaces with features such as self-cleaning, which can be useful in various applications such as those requiring waterproof and/or protective films. In this study, we prepared a solution from recycled polyethylene terephthalate (PET) and fabricated a superhydrophobic surface using electrospinning and electrospraying processes. We observed that the fabricated geometry varies depending on the solution conditions, and based on this, we fabricated a hierarchical structure. From the results, the optimized structure exhibited a very high WCA (>156.6°). Additionally, our investigation into the self-cleaning functionality and solar panel efficiency of the fabricated surface revealed promising prospects for the production of superhydrophobic surfaces utilizing recycled PET, with potential applications as protective films for solar panels. Consequently, this research contributes significantly to the advancement of environmentally friendly processes and the progress of recycling technology.

## 1. Introduction

Wettability is an important physical property that describes the interaction between a surface and a liquid. The wettability of a solid surface refers to the degree to which a liquid flows or spreads over it, which is determined by the interaction between the liquid molecules and the solid surface molecules. A surface is said to be “hydrophilic” if it spreads and wets easily with liquids, and “hydrophobic” if it impedes the spreading of liquids. The wettability of a surface is represented by its contact angle with the water. The surface is hydrophilic if the water contact angle is <90° and hydrophobic if it is ≥90°. At a WCA > 150°, the surface is considered superhydrophobic. Manipulating the wettability of surfaces has been used to solve a variety of problems in domains such as healthcare, energy, and the environment [1]. 

Classic examples of superhydrophobic surfaces can be found in nature. For example, the surface of a lotus leaf or taro leaf has a very small contact area with water droplets and slides easily. The leaf surface can repel solid particles, organic liquids, and biological contaminants through the rolling action of water droplets [2]. This phenomenon is known as the “lotus effect” and is attributed to the structural and chemical properties of the lotus leaf surface [3]. Superhydrophobic surfaces have contributed to many research advances over the last 30 years [4], as they have a wide range of functions for use in a variety of fields. For example, superhydrophobic surfaces can be applied to the exterior surfaces of automobiles, aircraft, and ships to reduce fluid drag and improve energy efficiency [5]. In addition, superhydrophobic coatings can be applied to the exterior walls or glass windows of buildings to give them self-cleaning ability [6], which helps to reduce maintenance and management costs. Superhydrophobicity can also be utilized in biomedicine, where coatings can be applied to the surfaces of medical devices to prevent the adhesion of viruses or bacteria, thus contributing to the prevention of infection [7]. Other functions include anti-icing [8], anti-corrosion [9], and anti-fog [10]. Recently, various techniques and materials have reportedly achieved superhydrophobicity. Superhydrophobic surfaces can be fabricated through the processes of surface roughing, electrospinning, lithography, deposition, coating, plasma treatment, and electrochemical synthesis [11]. Studies have been reported on the fabrication and utilization of hydrophobic surfaces based on electrospinning. For example, Qi et al. fabricated superhydrophobic surfaces and oil–water separators by growing ZnO on waste PET electrospinning fiber [12]. Jun et al. made superhydrophobic filters by making waste PET electrospinning fibers porous using N-methyl-2-pyrrolidone and acetone [13]. Both studies used recycled PET as the electrospinning material, but additional processes were performed with additional materials. These additional processes are mainly chemical coating processes, which have relatively high costs, pose environmental problems, and can be difficult to apply as they use complex methods. This paper presents a simple method for fabricating superhydrophobic surfaces based on electrospinning and electrospraying, without the need for additional processes. No materials are required except for the PET and a solution to dissolve it to fabricate the surface. 

Electrospinning and electrospraying are electrohydrodynamic processes involving the spinning or spraying of micro-/nanoscale structures by an electric field [14]. In electrospinning, a strong electric field is applied to a polymer solution, and the resulting Taylor cone at the tip of the syringe needle forms fibers with a diameter of micro-/nanoscale order on a charged collector [15]. Electrospraying is similar in principle. The main difference between the two processes is the different physical properties, such as viscosity and concentration, and type of solution. High-viscosity polymer solutions are used for electrospinning, while low-viscosity polymer solutions are used for electrospraying [16]. Electrospinning can produce extremely thin microfibers and is characterized by a high surface-area-to-volume ratio [17]. Electrospraying allows for the fabrication of nanoscale beads. As such, electrospraying has recently been applied in various fields such as drug delivery [18] and fuel cell or electrode catalysis [19,20]. It is also one of the most promising superhydrophobic surface fabrication techniques as it can efficiently fabricate surfaces with a high specific surface area relative to the volume [21]. Additionally, the process is fast and simple, and a wide range of nanoscale structures can be fabricated with a small amount of polymer solution. Therefore, electrospraying and electrospinning offer new solutions for superhydrophobic surface fabrication, which has been limited by cost and complexity. Superhydrophobic surfaces can be fabricated by generating micro-/nanoscale fiber and bead structures through electrospinning and electrospraying, respectively. Various structures can be produced by controlling conditions such as the polymer solution concentration, viscosity, voltage, flow rate, tip-to-collector distance, temperature, and humidity. Depending on the concentration of the polymer solution, the thickness of the fibers and the diameter of the beads produced by electrospinning and electrospraying vary [22]. Hierarchical structures with a large surface-area-to-volume ratio can be fabricated by adjusting these parameters.

Utilizing waste polyethylene terephthalate (PET) bottles to fabricate superhydrophobic surfaces is one of several promising approaches. PET, a lightweight low-cost polymer, has seen increasing use, leading to issues with its waste products [23]. Over the past 60 years, 12% of all plastic production has been incinerated, 60% has been discarded and landfilled, and only 9% has been recycled. It is estimated that 26,000 million metric tons (Mt) of PET waste will be generated in 2050, of which only 9000 Mt will be recycled [24]. PET is not biodegradable and releases toxic gases into the air during combustion [25]; thus, better management is needed to mitigate land and water pollution from PET. Our proposed approach offers a solution, as waste PET is used as the main material for our process. Recycling waste resources and transforming them into functional surfaces provides a sustainable solution while also responding to environmental issues. The fabrication of superhydrophobic surfaces by recycled PET electrospinning and electrospraying can find applications in various industrial fields, with the advantages of ecofriendliness, process simplicity, and economy.

This paper presents a novel method for fabricating superhydrophobic surfaces from waste PET bottles by utilizing electrospinning and electrospray coating techniques. We present an economical and simple solution to realize superhydrophobic surfaces using only a single recycled material. The effects of process parameters such as solution concentration and jet time on the morphology and wettability of the surface are investigated. Based on the investigated parameters, hierarchical structures are formed to create a superhydrophobic surface. Furthermore, we explore the self-cleaning capability and recyclability of the fabricated surfaces and offer practical applications based on their superhydrophobic properties. Through this research, we aim to contribute to the development of environmentally friendly and functional surfaces while also responding to PET waste.

## 2. Materials and Methods

This section presents the experimental materials, methods, and characterization measurements. Section 2.1 describes the materials used. Section 2.2 discusses the procedures and describes the equipment and software used to measure and analyze the experimental results.

### 2.1. Materials

**Figure 1:**
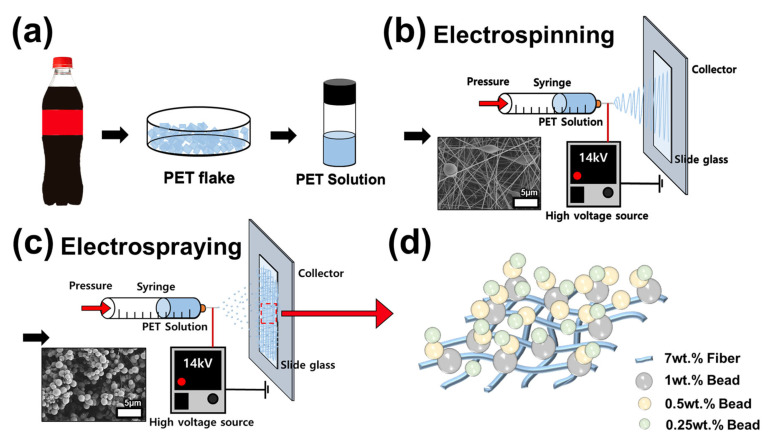
Schematic diagram of the fabrication process of a superhydrophobic surface. (**a**) Manufacturing process of the polymer solution using a waste polyethylene terephthalate (PET) bottle. (**b**) Electrospinning process of the waste PET solution. (**c**) Electrospraying process of the waste PET solution. (**d**) Schematic diagram of the final formation’s structure.

In this study, waste PET bottles were obtained from Coca-Cola (Charlotte, NC, USA) (Figure 1a). Dichloromethane (DCM; anhydrous, ≥99.8%, containing 40–150 ppm amylene as a stabilizer) and trifluoroacetic acid (TFA; ReagentPlus*^®^*, Old Bridge, NJ, USA, 99%) were purchased from Sigma Aldrich (St. Louis, MO, USA). All reagents were used without further purification.

### 2.2. Experimental Section

#### 2.2.1. Preparation of Waste PET Solution

TFA and DCM were mixed in a 3:1 ratio by weight. TFA and DCM have the advantage of high solubility in small amounts, making them suitable as solvents for PET [26]. Waste PET flakes produced from the Coca-Cola bottles were washed in deionized water and ethanol and then added to the solution according to the concentration. In this experiment, the concentration of recycled PET was determined as a weight percentage (wt.%) of the total solution weight. Four concentrations of recycled PET in the amounts of 7, 1, 0.5, and 0.25 wt.% were prepared. The mixed solution was sealed with parafilm and stirred in a vortex mixer for at least 8 h.

#### 2.2.2. Electrospraying and Electrospinning

Electrospinning and electrospraying processes were utilized to fabricate micro-/nanoscale surface structures. The 7 wt.% solution concentration was used for electrospinning, and 1, 0.5, and 0.25 wt.% solutions were used for electrospraying. Initially, the 7 wt.% solution was electrospun. Subsequently, 1, 0.5, and 0.25 wt.% solutions were then sequentially electrosprayed on top of the electrosprayed layer (Figure 1b,c). For hierarchy formation, fibers and beads were collected on a glass slide. Electrospinning was performed for 120 s, and electrospraying was performed for 600 s for all solution concentrations. A syringe equipped with a flat-end metal needle of outer diameter 0.642 and inner diameter 0.337 mm was used for flow control. The voltage was set to 14 kV, and the flow rate of the syringe pump was set to 0.01 mL/min. The-tip to-collector distance was set at 10 cm, the temperature was kept in the range of 18–21 °C, and the humidity was 31–33%. After the formation of the fiber surface using the 7 wt.% solution and electrospinning, the surface was dried completely in a convection oven, and then the beads were deposited on the fiber surface through a 1 wt.% solution electrospraying process to form a hierarchical structure. After another complete drying process, 0.5 wt.% solution electrospraying was performed. In the same way, after complete drying, a 0.25 wt.% solution was electrosprayed to complete the superhydrophobic surface formation. Through the above process, we aimed to fabricate superhydrophobic surfaces. Each fabricated surface was analyzed and applications were explored. 

#### 2.2.3. Self-Cleaning and Recyclability Tests

The fabricated surface was tested for self-cleaning function and surface recyclability. For the self-cleaning function test, graphite powder and DI water were used. The fabricated surface was positioned at an inclined angle, graphite powder was covered on the surface, and droplets of DI water were dripped through a syringe at a constant rate of 1 mL/min. The self-cleaning test was conducted by analyzing the water droplets rolling down the fabricated surface and being cleaned along with the impurities on the surface. The surface recyclability test proceeded as follows. First, the surface was fabricated by electrospinning for a longer period of time than the experimental conditions in order to obtain a sufficient amount of material. The fabricated surface was washed with DI water and dried completely. The dried surface was mixed with TFA and DCM at the weight ratio suggested in the experiment (7 wt.%) to prepare the solution. The solution was electrospun to produce the surface and compared with the recycled PET electrospun surface.

#### 2.2.4. Characterization

Field-emission scanning electron microscopy (FE-SEM; ULTRA PLUS; ZEISS, Oberkochen, Germany) was used to observe the morphology of the fibers and beads. The WCA and surface energy were measured using Smartdrop Plus (Femtobiomed, Inc., Gyeonggi-do, Republic of Korea), and the droplet size was 10 μL. The mechanical properties of the fiber and the bead thickness and diameter were measured using ImageJ software (version 1.53e, Java 1.8.0_172, Wayne Rasband, U.S. National Institutes of Health, Bethesda, MD, USA).

## 3. Results and Discussion

### 3.1. Morphology of the Electrospun and Electrosprayed Structures

**Figure 2:**
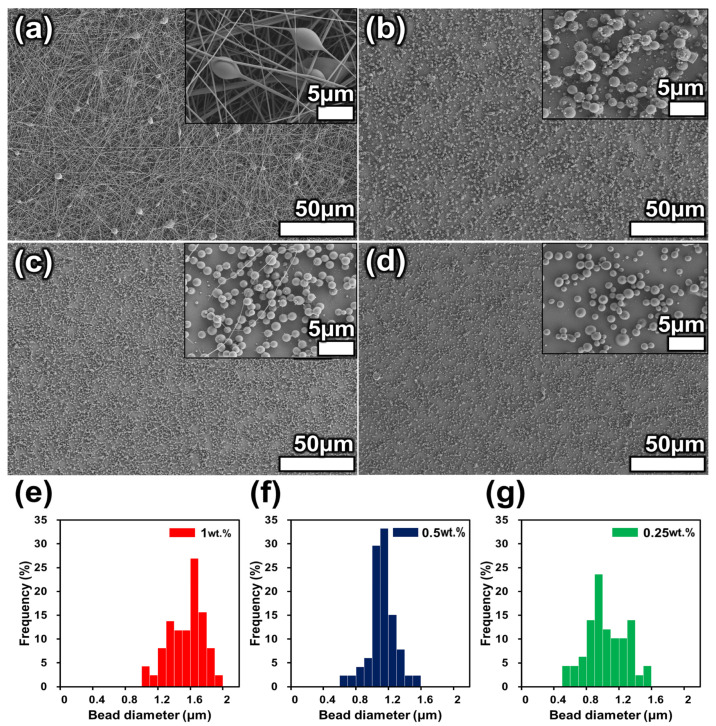
Scanning electron microscopy (SEM) images of fibers and beads produced by electrospinning and electrospraying as a function of the polymer solution concentration: (**a**) 7, (**b**) 1, (**c**) 0.5, and (**d**) 0.25 wt.%. Graphs of the bead diameter distribution as a function of the polymer solution concentration: (**e**) 1, (**f**) 0.5 and (**g**) 0.25 wt.%.

Depending on the concentration of the polymer solution, different structures were observed as a result of electrospinning and electrospraying. Figure 2 shows SEM images of the electrospun and electrosprayed solutions. Figure 2a shows a SEM image of the results from 7 wt.% solution electrospinning. Bead-on-a-string structures were observed along with nanoscale fibers. The bead-on-a-string structure showed a smaller contact area than the fiber surface, indicating a higher WCA [27]. These results were attributed to the Cassie–Baxter equation, which states that WCA increases as the fraction of contact area between liquid and solid decreases [28]. As measured by ImageJ software, fibers with a diameter of 0.229 ± 0.11 μm and beads with a diameter of 2.76 ± 0.7 μm were produced. Figure 2b shows a SEM image of the bead morphology resulting from electrospraying with a 1 wt.% polymer solution, which formed beads with an average size of 1.46 ± 0.2 μm. Figure 2c,d show the results obtained using 0.5 and 0.25 wt.% solution concentrations, respectively, which produced beads with average sizes of 1.04 ± 0.15 and 0.96 ± 0.27 μm, respectively. The average size of the beads produced changed with the polymer solution concentration. Fantini et al. confirmed that bead size decreases with decreasing solution concentration when electrospraying polystyrene, which is consistent with the results of this experiment with PET electrospraying [29]. Therefore, it is possible to fabricate a surface with a minimal contact area by mixing each of the fabricated structures. In the next section, a hierarchical superhydrophobic surface will be discussed.

### 3.2. Hydrophobicity Properties of the Hierarchical Surfaces

**Figure 3:**
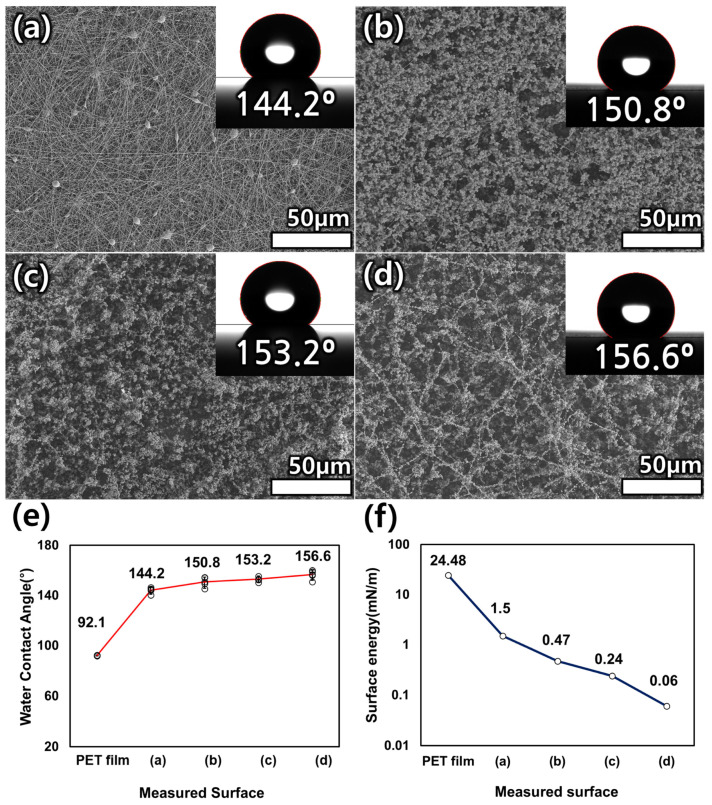
SEM images of the fabricated hierarchical surfaces and the water contact angle (WCA) for (**a**) 7, (**b**) 1, (**c**) 0.5, and (**d**) 0.25 wt.% waste PET on the ‘c’ surface. (**e**) Graph of the WCA (°) for each surface. (**f**) Graph of the solid surface energy (mN/m) for each surface.

Based on the structures described in the previous section, formation of a superhydrophobic hierarchical surface is described. The slide glass was sequentially electrospun and electrosprayed from a 7 wt.% polymer solution to a 0.25 wt.% solution. For each stage of the process, SEM images were taken and the WCA was measured. First, the 7 wt.% polymer solution was spun for 6 min (Figure 3a); fiber and bead-on-a-string structures were observed, and the WCA was measured to be 144.2°. This confirmed that the hydrophilic slide glass surface can be transformed into a hydrophobic surface by the electrospun fiber and bead-on-a-string structure. Figure 3b shows the surface created by electrospraying a 1 wt.% polymer solution of waste PET onto the surface created by the 7 wt.% solution. The WCA was 150.8°, an increase of 6.6° compared to before. This is due to the reduced contact area with water due to the generation of very small beads. An even smaller contact area can be expected if smaller beads are generated on this surface. Figure 3c shows the surface created by electrospraying a solution with a concentration of 0.5 wt.% waste PET on top of the surface in Figure 3b. The WCA increased by 2.4°–153.2° as a result of spraying smaller-diameter beads. Figure 3d shows the surface generated by electrospraying a 0.25 wt.% solution of waste PET over the surface shown in Figure 3c. By spraying the smallest-diameter beads, the contact area with water was minimized and a WCA of 156.6° was achieved. Figure 3e shows the WCA graphs of the PET surface and the fabricated surfaces in Figure 3a–d. The values of five repeated measurements of each surface were plotted as a scatter plot, and the mean value and standard deviation for each surface are shown. As a result, conversion of a hydrophobic surface with a WCA of 92.1° to a superhydrophobic surface with a mean of 156.6° is achieved. Also, reproducibility of the fabricated surface is confirmed, as the WCA shows a close difference in five repeated measurements. Figure 3f shows the solid surface energy of the smooth PET film and the fabricated surfaces in Figure 3a–d. The surface energy was measured using Smartdrop Plus (Femtobiomed, Inc., Republic of Korea). This device uses Young’s equation and the equation of state to derive the surface energy value, which is shown in the equation below.

(1) $$\cos\theta =-1+2\sqrt{\frac{\gamma_{sv}}{\gamma_{lv}}}e^{-\beta {\left(\gamma_{lv}-\gamma_{sv}\right)}^{2}}$$

In the above equation, θ is water contact angle. β was empirically calculated with a constant value of 0.0001247$\;{\left(m^{2}/\mathrm{mJ}\right)}^{2},\;$and the device measured the value of the liquid surface energy $\gamma_{lv}$ and calculated the solid surface energy $\gamma_{sv}$ [30]. DI water was used as the fluid for the measurement. In order to measure the surface energy of the same material as its morphology changed, both the surface of the PET film and the fabricated surfaces in Figure 3a–d were measured. The surface energy on the surface of the PET film decreased sharply after the generation of 7 wt.% fibers and gradually decreased in the subsequent process, finally changing from the initial 24.48 mN/m to 0.06 mN/m. This confirmed that the same material was processed to change the surface and create a surface structure with smaller units, resulting in a shape with lower surface energy. Additionally, the roughness coefficient of the fabricated surface was calculated based on the experimental results. Since the fabricated surface was very thin, it was calculated with Wenzel’s theory, which assumes no air entrapment by water droplets on the surface. The equations used are as follows [31].

(2) $$\cos\theta_{W}=r_{f}\cos\theta_{Y}$$

where $\theta_{w}$ is the Wenzel contact angle, $r_{f}$ is the roughness factor, which is always greater than 1, and $\theta_{Y}$ is the Young contact angle, which is the contact angle on a smooth surface made of the same material [32]. In the Wenzel state, for values of $r_{f}$ > 1, a hydrophobic surface has a larger contact angle, and a hydrophilic surface has a smaller contact angle. In other words, surface roughness always amplifies the inherent wettability of a surface [33,34]. The Young contact angle was characterized through thin films made by spin coating a solution of the same material as the fabricated surface. The fabricated film had a WCA of 92.1° and a $\cos\theta_{Y}$ value of −0.037, and the calculated values are shown in Table 1. 

Because the wetting state of the surfaces in each fabrication step is the Wenzel state as mentioned above, the roughness of the surface could be derived based on Equation (2). To accomplish the superhydrophobic surface with PET, the roughness has to be over 23.4. Even though electrospun PET fiber shows good modification, it cannot reach the superhydrophobic state, but the other hierarchical surfaces with the beads achieve that state. Moreover, the more complex the structure, the greater the contact angle. It was shown that piling beads with diminishing size on the electrospun fiber is effective.

### 3.3. Self-Cleaning Test

**Figure 4:**
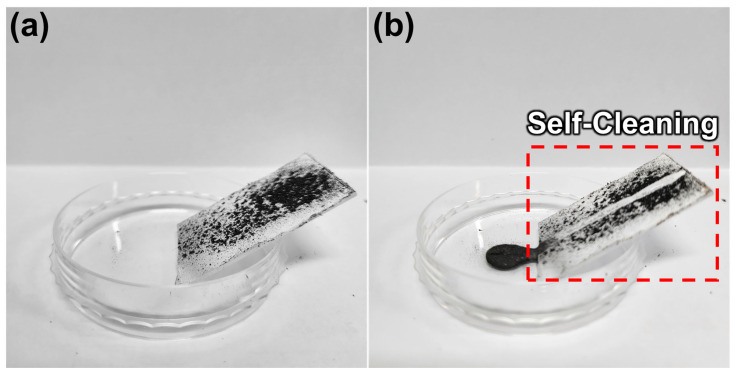
Self-cleaning function test results. (**a**) Fabricated superhydrophobic surface with impurities. (**b**) After dropping a few drops of water.

In this chapter, the self-cleaning ability of the fabricated superhydrophobic surfaces was tested. Self-cleaning is one of the most important properties of superhydrophobic surfaces. The fabricated surface was placed on an inclined surface and water droplets were placed on it to test its cleaning ability. The impurity was graphite powder. A large amount of graphite was placed on the fabricated surface (Figure 4a) and we rolled a few water droplets to check the self-cleaning ability (Figure 4b). After a large amount of water droplets were dropped onto the surface, the surface was completely cleaned. The experiment successfully confirmed the self-cleaning function of water droplets, which can easily roll on the surface and remove dirt. The ability of water droplets to easily remove impurities from a surface can be useful for self-cleaning coatings, waterproofing, etc. The self-cleaning capability confirmed in this experiment shows the potential for practical applications of the developed superhydrophobic surface. For example, in outdoor settings, such a surface could be utilized to prevent the accumulation of dirt, dust, and pollutants on various structures, ranging from solar panels to architectural facades [35,36]. It can also be used in transportation applications such as aerospace for reliability and efficiency [37,38]. 

### 3.4. Surface Recyclability Test 

**Figure 5:**
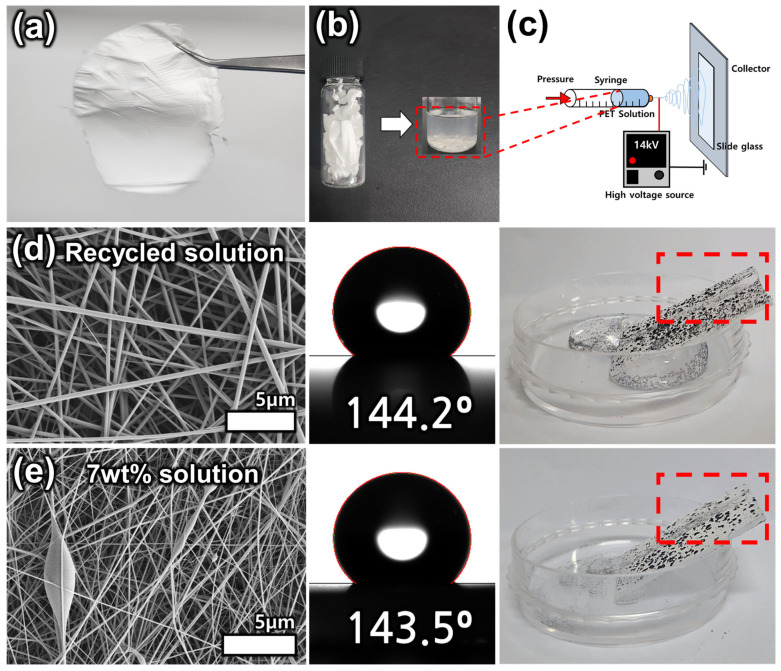
Recycling process of fabricated superhydrophobic surfaces: (**a**) Superhydrophobic surfaces, (**b**) polymer solution made by the fabricated surface, (**c**) fabricated surface recycling electrospinning process, (**d**) SEM, WCA images and self-cleaning test results of recycled surface and (**e**) 7 wt.% solution.

In this chapter, the recyclability of the fabricated surfaces was tested. Superhydrophobic surfaces acting as solutes were fabricated over a longer period of time to ensure the electrospinning solution volume. The fabricated superhydrophobic surface is first cleaned. After cleaning, the surface is redissolved in TFA and DCM (Figure 5b). The resulting polymer solution is then electrospun to produce fibers (Figure 5c). The recycled solution produced straight, smooth fibers (Figure 5d). It was also compared to a surface created with a 7 wt.% solution under the same condition (Figure 5d,e). Both surfaces produced ideal hydrophobic surfaces with a WCA ≥ 140°. Therefore, this reusable approach allows us to melt the fabricated superhydrophobic surface again and electrospun it, thus providing a sustainable solution for recycling plastic waste. However, for the self-cleaning test, it does not reach superhydrophobicity, showing a weak self-cleaning ability. This can be improved by the additional electrospraying process presented in this experiment. In conclusion, this method helps to reduce the consumption of additional materials for the formation of superhydrophobic surfaces, while also contributing to plastic waste management, thus expanding the application possibilities of superhydrophobic surfaces. There is also the potential to improve surface properties and impart various functionalities through the repeated electrospinning process. 

### 3.5. Application: Solar Panel Efficiency Test

**Figure 6:**
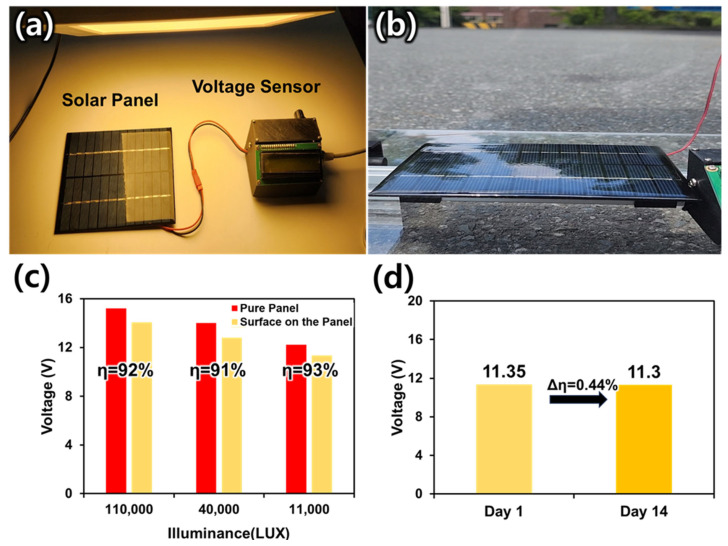
Device used in the experiment and test results. (**a**) Solar panels and voltage sensors used in the experiment. (**b**) Voltage measurements in natural light. (**c**) Graph of voltage produced by the solar panel and the efficiencies of the fabricated surfaces. (**d**) Panel efficiency with fabricated surfaces over time (days 1–14).

In this study, experiments were conducted to explore the potential for fabricated superhydrophobic surfaces to improve the performance of solar panels. Solar panel usage continues to increase worldwide [39]. However, the efficiency and lifespan of solar panels are greatly affected by surface contaminants, with efficiency losses as high as 15% [40]. Contamination on solar panels should be cleaned immediately, as it can block the sunlight they receive or corrode the surface. The fabricated superhydrophobic surface has self-cleaning ability, which means that it can remove impurities from its surface. To verify its applicability, an Arduino-controlled solar panel voltage test device was created, and an efficiency measurement experiment was conducted (Figure 6a). In the experiment, the voltage generated by light in the same circumstance was measured by comparing a pure panel with nothing on top of the panel with a panel covered with a superhydrophobic surface. To measure the voltage in different situations, experiments were conducted in bright natural light (i.e., in broad daylight), dim natural light in the morning, and artificial light (Figure 6a,b). The surface was fabricated on the thin glass to match the size of the solar panel and the voltage was measured with all parts covered. All conditions were kept constant except for illumination and fabricated surface placement. The results showed that in broad daylight (110,000 lux), the voltage of the pure solar panel was 15.21 V, the voltage of the panel covered by the fabricated surface was 14.06 V, and the efficiency was 92%. In natural light in the morning (40,000 lux), the pure solar panel had a voltage of 14.01 V, and the panel covered by the fabricated surface has a voltage of 12.81 V and an efficiency of 91%. Under artificial light (11,000 lux), the voltage produced by the pure solar panel was 12.23 V and that of the panel covered by the fabricated surface was 11.35 V, with an efficiency of 93%. Under different illumination conditions, the efficiency remained at 91%. Plus, to test the stability of the efficiency protection of surfaces built over a period of time, the efficiency was measured after 14 days under the same conditions. For illumination control, the experiments were conducted under artificial light at 11,000 lux. From before to after 14 days, the voltage decreased from 11.35% to 11.3%, with a change rate of 0.44%. This is within the margin of error, so it is concluded that the manufactured panel shows a consistent performance for long-term use. These experimental results suggest the possibility of utilizing the fabricated superhydrophobic surfaces as solar-panel-protective films. Fabricated superhydrophobic surfaces can help maintain and protect solar panels by preventing the ingress of contaminants, dust, and other impurities from the outside through their self-cleaning capabilities. Superhydrophobic surfaces can also improve the productivity of solar panels by maintaining voltage. Furthermore, the current efficiency is expected to be further improved through optimal condition studies or surface modification studies. 

## 4. Conclusions

In this study, we successfully developed an ecofriendly method to fabricate superhydrophobic surfaces using recycled PET through electrospinning and electrospraying processes without additional processing. Based on a fundamental understanding of wettability and superhydrophobicity, we aimed to produce superhydrophobic surfaces while recycling waste PET. The concentration of the solution was controlled by varying the amount of recycled PET flakes in the polymer solution. We observed and precisely analyzed how the structure produced varied depending on the solution concentration. By creating a hierarchical structure with these micro-/nanoscale structures, we minimized the contact area with water droplets and produced a surface with superhydrophobic properties. As a result, we achieved a very high WCA (>150°) in the optimized fabrication condition, which is significant in that we succeeded in producing a superhydrophobic surface using only nanostructures and without additional treatment processes such as chemical coatings. The performance of the fabricated film was verified through self-cleaning tests and solar panel application experiments, and excellent self-cleaning and efficiency protection of about 92% were confirmed, which can be improved through further research. This research enables the fabrication of environmentally friendly superhydrophobic surfaces, as an alternative to polymer waste, where the fabricated surface can be used to produce new films while recycling waste polymers such as PET.

## Figures and Tables

**Table 1:** Roughness factors and substituted values for fabricated superhydrophobic surfaces obtained from Wenzel’s theory.

Surface	$\theta_{w}$	$\cos\theta_{W}$	$\cos\theta_{Y}$	$r_{f}$
(a)	144.2	−0.81	−0.037	21.92
(b)	150.8	−0.87		23.57
(c)	153.2	−0.89		24.14
(d)	156.6	−0.92		24.81

## Data Availability

The datasets generated during and/or analyzed during the current study are available from the corresponding author on reasonable request.
